# Use of sugammadex in a patient with progressive muscular atrophy and in a patient with amyotrophic lateral sclerosis: Case report

**DOI:** 10.1097/MD.0000000000007098

**Published:** 2017-06-08

**Authors:** Jae Hwa Yoo, Soon Im Kim, Sun Young Park, Mi Roung Jun, Yong Eun Kim, Hyoung June Kim

**Affiliations:** Department of Anesthesiology and Pain Medicine, Soonchunhyang University Seoul Hospital, Seoul, Korea.

**Keywords:** amyotrophic lateral sclerosis, progressive muscular atrophy, sugammadex

## Abstract

**Introduction:**

We herein present 2 cases involving the combination of rocuronium and sugammadex in patients with motor neuron disease. The patients were a 54-year-old man with progressive muscular atrophy who underwent removal of internal fixators in the arm and leg, and a 66-year-old woman with amyotrophic lateral sclerosis who underwent skin grafting in the left lower leg. General anesthesia was induced with propofol, rocuronium, and remifentanil and maintained with desflurane and remifentanil. At the end of the surgical procedure, we administered sugammadex. Three or 4 minutes after administration of sugammadex, the patients began to breathe spontaneously and were extubated without complications.

**Conclusion:**

Sugammadex can be used successfully to reverse neuromuscular blockade in patients with motor neuron disease.

## 1 Introduction

Patients with motor neuron diseases who are treated by surgery under general anesthesia are subjected to perioperative risks including respiratory complications, which are the most common causes of death in such patients. Clinicians must be particularly careful when using neuromuscular blockade in these patients. Succinylcholine is contraindicated because of lethal hyperkalemia, and nondepolarizing neuromuscular blocking agents have shown increased sensitivity in patients with motor neuron disease.^[1,2]^ They are vulnerable to the risks of postoperative residual curarization, respiratory complications.

Sugammadex is used for selective reversal after administration of an amino-steroidal neuromuscular blockade agent.^[3]^ It encapsulates rocuronium and is rapidly excreted in the urine.^[3]^ Sugammadex has several advantages, including rapid recovery and avoidance of postoperative residual curarization.^[3]^ This feature of sugammadex may make it suitable for patients with motor neuron disease.

Amyotrophic lateral sclerosis (ALS) is the most common motor neuron disease that affects both upper and lower motor neurons.^[4]^ Progressive muscular atrophy (PMA) is a rare and sporadic motor neuron disorder characterized by clinically isolated lower motor neuron degeneration.^[5]^ A few case reports about the use of sugammadex in patients with ALS have been published.^[6–8]^ In addition, no case reports have described the use of sugammadex in patients with PMA with bulbar symptoms.

We herein present 2 cases involving the successful use of rocuronium and sugammadex during anesthesia in a patient with ALS and another with PMA.

## 2 Case descriptions

Consent for publication of this report was obtained from our patients. However, ethical approval was not necessary for this case report in our institution, because it does not provide the institutional review board number for case reports.

### 2.1 Case 1 PMA

A 54-year-old man (weight, 70 kg; height, 175 cm) was scheduled for removal of an intramedullary nail from the left femur and plate from the left humerus because of pain. He had been diagnosed with PMA at 16 years of age, as evidenced by weakness in both legs and progressive weakness and atrophy of the lower and upper extremities. He had undergone insertion of an intramedullary nail into the left fractured femur and insertion of a plate into the left fractured humerus at 42 years of age. At that time, he had no bulbar symptoms, and his motor grade was ±4 in both the upper and lower extremities. These operations had been performed under general anesthesia (O_2_, N_2_O, sevoflurane, and vecuronium) without complications.

Upon admission to our institution, he showed excessive muscle weakness and atrophy with noticeable fasciculation of both the upper and lower extremities. His motor grade was 1 to 2 in the left extremities and 3 in the right extremities. His deep tendon reflex was decreased and pathologic reflex was absent. Additionally, his tongue was atrophied and he had mild dysphagia and dysarthria. A chest X-ray showed diffuse pleural thickening and a hypertensive heart contour. Findings of a cardiologic examination, including electrocardiography (ECG), echocardiography, and laboratory tests, were unremarkable.

The patient received 0.2 mg of glycopyrrolate intramuscularly for premedication 30 minutes before the operation. Upon arrival in the operating room, we applied standard monitoring, reactive and stable entropy, and neuromuscular monitoring. The patient's initial blood pressure was 170/86 mm Hg, heart rate was 111 beats/min, SpO_2_ was 93%, and response and state entropy was 98/96.

General anesthesia was induced using 150 mg of 1% propofol with 40 mg of lidocaine after preoxygenation with 100% oxygen. After induction, we began to monitor the train-of-four (TOF) ratio, which was 113% at supramaximal stimulus. We then administered 30 mg of rocuronium (0.43 mg/kg) and started continuous infusion of remifentanil (0.05–0.5 μg/kg/min). Two minutes after rocuronium administration, his TOF count was 0, and we intubated his trachea without difficulty. Anesthesia was maintained with desflurane at 3 to 4 vol% and remifentanil at 0.01 to 0.1 μg/kg/min, with a target response and state entropy of 40 to 50. His vital signs and TOF ratio were measured every 5 minutes until completion of surgery. Two hours after starting anesthesia, his TOF ratio recovered to 15%, and we administered an additional 5 mg of rocuronium because of fasciculation of his operating arm. His vital signs remained stable during the surgery.

The surgery was completed 2 hours 40 minutes after starting anesthesia. Five minutes before the end of surgery, we discontinued the remifentanil and administered 50 μg of fentanyl. At the end of the surgery, his TOF ratio was 15%, and we administered 200 mg of sugammadex (2.86 mg/kg) and discontinued the desflurane. One minute after sugammadex administration, his TOF ratio recovered to >0.9. After an additional 2 minutes, his TOF ratio was 125%, his tidal volume had recovered, and he could open his eyes and follow our commands. Tracheal extubation was performed with active suctioning of oral secretions. After extubation, he was able to breathe spontaneously without difficulty, and his SpO_2_ remained at 99% with 6 L/min of 100% oxygen via a face mask. We then transferred the patient to the postanesthesia care unit, where he complained of a dry mouth and pain at the operation site. We administered an additional 50 μg of fentanyl. After 50 minutes, his SpO_2_ remained at 98% to 99% on room air without any signs of respiratory complications. He was then transferred to the general ward. He had no respiratory complications or worsening of his neurologic signs/symptoms during the postoperative period.

### 2.2 Case 2 ALS

A 66-year-old woman (weight, 40 kg; height, 154 cm) was scheduled for split-thickness skin grafting from the left lateral thigh to the left lower leg because of a traumatic skin defect. She was diagnosed with ALS in another hospital 18 months previously, as evidenced by dysarthria, dysphagia, and motor weakness of both legs.

The patient presented with progressive weight loss, general weakness, fatigue, tongue atrophy, progressive dysphagia, and dysarthria. Her motor grades in the right extremities and left extremities were 2 and 3, respectively, and she exhibited fasciculation of her extremities. A chest X-ray showed subsegmental atelectasis. The results of a cardiologic examination, including ECG, echocardiography, and laboratory tests, were unremarkable.

The patient received glycopyrrolate (0.1 mg) intramuscularly for premedication 30 minutes before the operation. Upon arrival in the operating room, standard monitoring (noninvasive blood pressure, ECG, SpO_2_), bispectral index, and neuromuscular monitoring was commenced. The patient's initial blood pressure was 143/76 mm Hg, heart rate was 83 beats/min, SpO_2_ was 93%, and bispectral index was 93.

General anesthesia was induced using 70 mg of 1% propofol with 40 mg of lidocaine after preoxygenation with 100% oxygen. We then began to monitor the TOF ratio. At that time, the patient's TOF ratio was 125% at supramaximal stimulus. We then administered 20 mg of rocuronium (0.5 mg/kg) and started continuous infusion of remifentanil (0.05–0.5 μg/kg/min). Two minutes after rocuronium administration, the patient's TOF count was 0 and we intubated her trachea without difficulty. Anesthesia was maintained with desflurane at 3 to 4 vol% and remifentanil at 0.01 to 0.1 μg/kg/min, with a target bispectral index of 40 to 50. Her vital signs and TOF ratio were measured every 5 minutes until completion of surgery. Additional doses of rocuronium were not needed because her TOF count was maintained at 0 until the end of surgery. Additionally, her vital signs remained stable during the surgery.

The surgery was complete 1 hour after starting anesthesia. Five minutes before the end of surgery, we discontinued the remifentanil and administered 50 μg of fentanyl. At the end of surgery, the TOF count was still 0; therefore, we administered 200 mg of sugammadex (5 mg/kg) and discontinued the desflurane. Two minutes after sugammadex administration, her TOF ratio recovered to >0.9. After an additional 2 minutes, her TOF ratio was 115% and she was able to breathe spontaneously, open her eyes, and follow our commands. Tracheal extubation was then performed with active oral secretion suctioning. After extubation, she was able to breathe spontaneously without difficulty and her SpO_2_ remained at 98% with 6 L/min of 100% oxygen via a face mask. We transferred her to the postanesthesia care unit, where she complained of pain at the operation site. We administered an additional 25 μg of fentanyl. After 30 minutes, her SpO_2_ remained at 98% to 99% on room air without any signs of respiratory complications. She was then transferred to the general ward. She had no respiratory complications or worsening of her neurologic signs/symptoms during the postoperative period.

## 3 Discussion

ALS is the most common of all motor neuron diseases affecting both upper and lower motor neurons, and its reported incidence is 1 to 2 per 100,000 people.^[4]^ The clinical symptoms of ALS are muscle weakness, atrophy, fasciculations, spasticity, and hyper-reflexia. Approximately 50% of patients with ALS die within 3 years of disease onset, and the most common cause of death is respiratory failure.^[4,9]^

PMA is a rare and sporadic motor neuron disorder characterized by clinically isolated lower motor neuron degeneration.^[5]^ It reportedly constitutes 2.5% to 11.0% of all motor neuron diseases.^[5]^ Patients with PMA have been considered to have longer expectancy than patients with ALS.^[5]^ However, many researchers have asserted that PMA is a lower motor neuron–prominent form of ALS, because a substantial number of patients diagnosed with PMA eventually develop upper motor neuron symptoms (spasticity, hyper-reflexia, and preserved tendon reflexes and pathologic reflexes), and some patients without clinical upper motor neuron symptoms exhibit upper motor neuron pathology at autopsy.^[5]^ In addition, many researchers have stated that the progression of PMA does not differ from that of ALS.^[10]^

The prognosis of ALS is associated with patient age at onset, disease site at onset, and pulmonary function.^[9]^ The prognostic factors for PMA do not differ from those for ALS.^[10]^ In our patient with ALS, the symptoms first began at 64 years of age and affected the bulbar regions; the disease then progressed rapidly. In our patient with PMA, however, the symptoms first began at 16 years of age and did not affect the bulbar regions at onset; thereafter, the disease exhibited slow progression. However, after the 26 years of disease progression, at the time of surgery, the patient had bulbar symptoms (tongue atrophy, mild dysphagia, and dysarthria). We recommended pulmonary function testing for respiratory assessment, but both patients refused the test because of the difficulty of performing it. We determined that the risk is high on the basis of clinical status, although not performed pulmonary function test.

Regional anesthesia is used because it avoids the risk of respiratory complications.^[11]^ However, the risk of demyelinating nerve injury due to direct needle trauma and neurotoxicity of local anesthetics cannot be ignored.^[12]^ General anesthesia without muscle relaxants has been successfully used in patients who do not require deep muscle relaxation.^[13]^ Minimal doses of muscle relaxants have been used with careful monitoring in some patients who require deep muscle relaxation.^[14]^ However, the risks of postoperative residual curarization and respiratory complication remain. Therefore, we decided to perform general anesthesia using rocuronium and sugammadex, because the fasciculation in our patients would have made the operation difficult to perform.

Some recent reports have described the successful use of rocuronium and sugammadex in patients with ALS. Kelsaka et al^[8]^ performed general anesthesia using rocuronium and sugammadex for plate fixation of a humeral neck fracture. They used 20 mg (0.28 mg/kg) of rocuronium for intubation and an additional 10 mg of rocuronium for maintenance. At the end of surgery, they used 2 mg/kg of sugammadex for reversal of neuromuscular blockade without any complications. Karaoglan et al^[7]^ used 0.6 mg/kg of rocuronium for percutaneous endoscopic gastrostomy because of difficult mouth opening in a patient with a tracheostomy and 2 mg/kg of sugammadex at the end of the procedure without any complications. Chang et al^[6]^ also used 0.3 mg/kg of rocuronium for intubation and an additional 10 mg of rocuronium because of bucking fasciculation, and 1 mg/kg of sugammadex was used for reversal of neuromuscular blockade without complications. In addition, the use of sugammadex in other lower motor neuron disease has been reported. Takeuchi et al^[15]^ used 40 mg of rocuronium for intubation, and 2 mg/kg of sugammadex was used for reversal of neuromuscular blockade without any complications in a patient with spinal and bulbar muscular atrophy which is X-linked recessive lower motor neuron disease. However, no reports have described the use of sugammadex in patients with PMA with bulbar symptoms.

In our patients, a reduced dosage of rocuronium (ALS case, 0.5 mg/kg [20 mg]; PMA case, 0.43 mg/kg [30 mg]) was needed for tracheal intubation. No additional doses of rocuronium were needed in the patient with ALS, and only 5 mg of rocuronium was used for intraoperative maintenance in the patient with PMA. We administered sugammadex before TOF recovery at the end of the surgery (ALS case: TOF count of 0, 5 mg/kg of sugammadex used; PMA case: TOF ratio of 15%, 2.86 mg/kg of sugammadex used). At 3 to 4 minutes after administration of sugammadex, they fully recovered without complications.

In conclusion, we have herein described the successful management of general anesthesia using rocuronium and sugammadex in 2 patients with motor neuron disease. The combination of rocuronium and sugammadex has been used successfully in patients with other rare motor neuron diseases with bulbar symptoms (eg, PMA) as well as in patients with ALS. The use of rocuronium and sugammadex is thought to be useful in the management of general anesthesia in patients with motor neuron diseases.
