# Use of sugammadex in a patient with progressive muscular atrophy and in a patient with amyotrophic lateral sclerosis: Case report: Erratum

**DOI:** 10.1097/MD.0000000000007339

**Published:** 2017-06-23

In the article, “Use of sugammadex in a patient with progressive muscular atrophy and in a patient with amyotrophic lateral sclerosis: Case report”,^[1]^ which appeared in Volume 96, Issue 23 of *Medicine*, the funding information, “This work was supported by the Soonchunhyang University Research Fund,” is missing.
